# Chemical Composition and Seasonality of Aromatic Mediterranean Plant Species by NMR-Based Metabolomics

**DOI:** 10.1155/2015/258570

**Published:** 2015-02-16

**Authors:** Monica Scognamiglio, Brigida D'Abrosca, Assunta Esposito, Antonio Fiorentino

**Affiliations:** Department of Environmental Biological and Pharmaceutical Sciences and Technologies, Second University of Naples, Via Vivaldi 43, 81100 Caserta, Italy

## Abstract

An NMR-based metabolomic approach has been applied to analyse seven aromatic Mediterranean plant species used in traditional cuisine. Based on the ethnobotanical use of these plants, the approach has been employed in order to study the metabolic changes during different seasons. Primary and secondary metabolites have been detected and quantified. Flavonoids (apigenin, quercetin, and kaempferol derivatives) and phenylpropanoid derivatives (e.g., chlorogenic and rosmarinic acid) are the main identified polyphenols. The richness in these metabolites could explain the biological properties ascribed to these plant species.

## 1. Introduction

Aromatic plants are widespread throughout the world and they are extensively added to different food preparations. The use of these plants is very popular and has a long tradition in Mediterranean area [1].

Plants in general have been shown to produce a wide range of chemicals, traditionally categorized into primary and secondary metabolites. For the sake of simplicity, primary metabolites can be thought of to serve nutritional purposes, while secondary metabolites are required by plants as weapons against competitors, herbivores or pathogens, and so forth [2]. However, both classes of metabolites are important for the plant itself but also for their actions on plant consumers and mainly in case of edible plants.

Many aromatic plants are added to foods and eaten. The whole plants or one or some of their components are used as, for example, food preservatives, flavour, and additives. Nevertheless, it has been shown that chemical composition of plants is highly variable along the year. Several analytical techniques are available for studying plant metabolites' content. Most of them are targeted techniques as an* a priori* knowledge on the metabolites to be analysed is required [2, 3]. Furthermore, for aromatic plants, a great effort has been devoted to the study of essential oils [2, 4].

Given this background, a wider knowledge about their whole metabolite content is needed. To this end, a very powerful approach is metabolomics, the comprehensive analysis of the set of low molecular weight compounds of a biological system under a given condition [5]. Analogously, other related approaches, like metabolic profiling [6], could be used.

In particular, NMR-based metabolomics has been shown to be very useful due to its untargeted and unbiased features [7]. Furthermore, it is highly reproducible, it allows the contemporary identification and quantification of a large number of compounds and needs short times of analysis (including the extraction procedures) [6]. The only limitation of NMR is its low sensitivity when compared to mass spectrometry, although sensitivity has been drastically increased with recent advances like higher magnetic fields and the introduction of microcryoprobes [5, 8]. On the other hand, NMR allows the identification of unknown compounds in the analysed mixtures, as it gives important structural information [8].

In order to demonstrate the potentiality of this approach, it has been applied to seven aromatic plant species characteristic of Mediterranean* garrigue*: the metabolites' content of these plants has been determined and the seasonality of their accumulation has been studied.

## 2. Materials and Methods

### 2.1. Plant Material Sampling and Processing

Seven plant species (Table 1) were collected in a* garrigue* on the calcareous hills of Durazzano, (41°3′N, 14°27′E; southern Italy) in winter (February 2012), spring (May 2012), summer (July 2012), and autumn (October 2012). The plants were selected based on their occurrence in the study site.* Origanum vulgare* samples were not available in autumn. Plant leaf samples were collected in the field always at the same moment of the day, in order to minimize differences due to metabolites changing based on circadian clock.

Three leaf samples (biological replicates) of each plant species were harvested and immediately frozen in liquid N_2_ in order to avoid unwanted enzymatic reactions and stored at −80°C up to the freeze drying process. Once freeze dried they were powdered in liquid nitrogen and stored at −20°C. Each sample was extracted and analysed by NMR.

Voucher specimens for all the plant species were deposited at the herbarium of the Second University of Naples (Table 1).

### 2.2. Metabolomics Analysis

Freeze-dried plant material (50 mg) was transferred to a 2 mL microtube. NMR samples were prepared in a mixture of phosphate buffer (Fluka Chemika; 90 mM; pH 6.0) in D_2_O (Cambridge Isotope Laboratories) containing 0.1% w/w trimethylsilylpropionic-2,2,3,3-*d*_4_ acid sodium salt (TMSP, Sigma-Aldrich) and methanol-*d*_4_ (Sigma-Aldrich). A volume of 1.5 mL of phosphate buffer and methanol-*d*_4_ (1 : 1) was added to the plant samples. The mixture was vortexed at room temperature for 1 min, ultrasonicated (Elma Transonic Digitals) for 40 min, and centrifuged (Beckman Allegra 64R) at 13000 rpm for 10 min. An aliquot of 0.6 mL was transferred to an NMR tube and analysed by NMR [9]. NMR spectra were recorded at 25°C on a 300.03 MHz for ^1^H and 75.45 MHz for ^13^C on a Varian Mercury Plus 300 Fourier transform NMR. CD_3_OD was used as the internal lock. Each ^1^H NMR spectrum consisted of 256 scans with the following parameters: 0.16 Hz/point, acquisition time (AQ) = 1.0 s, relaxation delay (RD) = 1.5 s, 90° pulse width (PW) = 13.8 *μ*s. A presaturation sequence was used to suppress the residual H_2_O signal. Free induction decays (FIDs) were Fourier transformed with LB = 0.3 Hz and the resulting spectra were manually phased and baseline-corrected and calibrated to TMSP at 0.0 ppm, using ^1^H NMR processor (MestReNova, version 8.0.2).

### 2.3. Quantitative Analysis

The main metabolites identified in plant extracts were analyzed by quantitative analysis. ^1^H-NMR spectra were bucketed, reducing it to integral segments with a width of 0.02 ppm with ACDLABS 12.0 ^1^H-NMR processor (ACDLABS 12.0, Toronto, Canada). Spectra were scaled to the internal standard (whose area, from −0.01 to 0.01 ppm, was set equal to 1). For each metabolite, buckets corresponding to nonoverlapping signals were used to calculate the relative amount as follows:

(1) $$\begin{array}{c} \text{Metabolite}\;\text{relative}\;\text{amount}=\frac{\text{SA}\times n_{\text{HTMSP}}}{n_{S}}, \end{array}$$

where SA is the metabolite signal area, but it is also equal to the signal area/standard area ratio, as standard area is equal to 1; *n*_HTMSP_ is a constant equal to 9 (the number of protons responsible for the signal between −0.01 and 0.01 ppm) and *n*_*s*_ is the number of protons of the metabolite signal area [10].

## 3. Results and Discussion

Recent research has shown culinary herbs and spices as a source of bioactive compounds [11]. Although most of them have been extensively studied for their essential oil composition, far less information is available on their polar and semipolar chemical composition.

**Figure 1:**
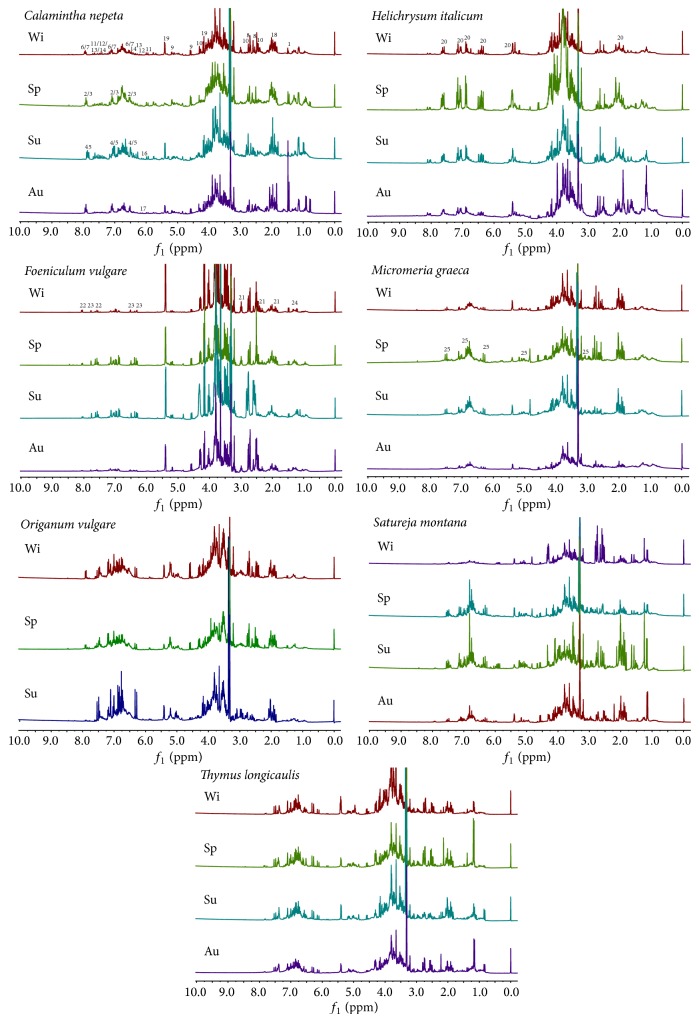
^1^H-NMR spectra of studied plants (Au = autumn; Sp = spring; Su = summer; Wi = winter). The main resonances of the main compounds are indicated on the spectra as follows: 1, alanine; 2, apigenin derivative 1; 3, apigenin derivative 2; 4, apigenin derivative 3; 5, apigenin derivative 4; 6, apigenin derivative 5; 7, apigenin derivative 6; 8, citric acid; 9, glucose; 10, malic acid; 11, phenylpropanoid 1; 12, phenylpropanoid 2; 13, phenylpropanoid 3; 14, phenylpropanoid 4; 15, phenylpropanoid 5; 16, phenylpropanoid 6; 17, phenylpropanoid 7; 18, quinic acid; 19, sucrose; 20, chlorogenic acid; 21, GABA; 22, kaempferol; 23, quercetin; 24, threonine; 25, rosmarinic acid.

Herewith, seven Mediterranean plants (*Calamintha nepeta*,* Helichrysum italicum*,* Foeniculum vulgare*,* Micromeria graeca*,* Origanum vulgare*,* Satureja montana*, and* Thymus longicaulis*) have been studied for their metabolite content by NMR. The identification of metabolites was carried out by comparing NMR data with an in-house library, with databases [12], and with some literature data [10, 13–15]. ^1^H-NMR data and extract composition are given in Table 2 and spectra are shown in Figure 1.

Primary metabolites were easily identified based on data extensively reported in literature of spectra acquired in the same solvent mixture [10, 12–15]. Among free amino acids, alanine was observed in all of the plants, while threonine was only detected in* Micromeria graeca* and* Foeniculum vulgare*.

The sugar content was highly variable, with glucose and sucrose as the main free carbohydrates detected.

Finally, some organic acids were identified. Quinic acid was present in all of the plants but* Helichrysum italicum* and* Satureja montana*, while malic acid was clearly detected in all of the plants.

Concerning the secondary metabolite content, the analysed Lamiaceae plants were all characterized by the presence of high amounts of rosmarinic acid (with the exception of* C. nepeta*), along with analogous compounds. Caffeic acid was identified based on comparison of NMR data with the literature [10, 15] and confirmed by comparison with NMR spectra of an in-house library. Rosmarinic acid was identified based on the comparison with already reported data [10] and the structure was confirmed by 2D NMR analysis. Indeed, the olefinic proton at *δ* 7.50 (H7) (showing HSQC correlation with the carbon at *δ* 145.9) and that at *δ* 6.30 (showing HSQC correlation with the carbon at *δ* 114.3) showed long range correlations with a carbon at *δ* 168.4 (C9). This carbon was in turn correlated with the proton at *δ* 5.02 (H8′), confirming the linkage between a caffeoyl moiety and the 3,4-dihydroxyphenyl lactic acid moiety. Furthermore, the former was identified based on the long range correlation of the H7 olefinic proton with the aromatic carbon at *δ* 126.4 (C1), showing further correlations with the signals belonging to an ortho/para trisubstituted aromatic ring (Table 2). The latter was identified as follows: the proton H8′ showed correlations with a carboxylic carbon at *δ* 176.5 and with a methylene carbon at *δ* 36.9 (C7′), showing HSQC correlations with the diastereotopic protons H7′ (Table 2). The proton H8′ also showed long range correlation with a quaternary aromatic carbon at *δ* 130.0 (C1′), in turn correlated with the signals belonging to a second ortho/para trisubstituted aromatic ring (Table 2).

Some phenylpropanoids in the extracts were not definitively characterized, inasmuch as, based on their scarce abundance and/or strong signal overlapping, they did not show clear correlations in 2D NMR spectra. However, the characteristic signals and correlations of the* trans*-propenylic chain suggested their presence. Indeed, correlations were observed in the HSQC, among the olefinic signals with carbons at 140–145 ppm (for the proton at lower fields) and at 114–120 ppm (for the proton at higher fields) and long range correlations were shown with carbon resonances attributable to ester carboxyl carbons and with quaternary aromatic carbons.

*Calamintha nepeta* extracts were also rich in several flavonoids and phenylpropanoids. Unfortunately, it was not possible to definitely characterize these compounds, but all of the flavonoids were identified as apigenin derivatives (Table 2). Indeed, several sets of resonances attributable to meta coupled protons H7/H8 (ring A), to proton H3 and to B ring ortho coupled protons were detected. Interestingly, the compounds, probably characterized by a different degree of glycosylation, showed a peculiar distribution along the seasons. Two apigenin derivatives were detected in spring and autumn (apigenin derivatives 1 and 2), while two different couples of these compounds were detected in summer (apigenin derivatives 3 and 4) and winter (apigenin derivatives 5 and 6) samples. Moreover, apigenin derivatives 5 and 6 were detected only in winter also in* Satureja montana*.

Analogously, as shown in Table 2, the presence of some phenylpropanoids was strongly dependent on the collection season (Table 2): phenylpropanoid 2 was detected only in winter, phenylpropanoid 5 only in spring, phenylpropanoid 6 only in summer, and phenylpropanoid 7 only in autumn samples.

The most stable metabolome along the seasons was detected for* Thymus longicaulis* while* Micromeria graeca* and* Origanum vulgare* only changed for some metabolites. However, differences in the amounts of the compounds were observed. Indeed, for all the Lamiaceae plants, a higher amount of aromatic compounds (Table 2) was observed in spring and summer samples compared to autumn and winter samples.* Helichrysum italicum* and* Foeniculum vulgare* extracts showed an analogous behaviour, with changes of metabolites mainly on the quantitative point of view.

*Helichrysum italicum* extracts, besides chlorogenic acids, also showed signals attributable to a 3-hydroxybenzofuran and an isobenzofuranone derivative. Chlorogenic, neochlorogenic, and dicaffeoylquinic acids were identified based on comparison of ^1^H-NMR data with the literature [10, 15] and with the in-house library. The caffeoyl moiety was clearly identified based on 1D and 2D NMR data and the linkage(s) with the quinic acid moiety was confirmed by the correlation observed in the long range spectrum. The 3-hydroxybenzofuran and isobenzofuranone derivatives were identified based on comparison with the NMR spectrum of the compound previously isolated [16].

Finally,* Foeniculum vulgare* was characterized by chlorogenic acids and flavonoids, identified, based on ^1^H-NMR data as kaempferol and quercetin [17]. Chlorogenic acid was reported for the first time from this species, to the best of our knowledge.

The identification of water soluble compounds in these plants is very important as most of them are added to dishes; hence they might be eaten or however they could release bioactive compounds into food. In this framework, it is worth to underlining that first of all the health promoting capacity of bioactive compounds could be dependent on synergisms. Secondly, these plants could also contain potential toxic compounds, as reported for several Lamiaceae [17]. Nevertheless, the role in nutrition of primary metabolites is often disregarded [18]. These considerations raise the attention to the need for a comprehensive profiling of their metabolites.

Furthermore, the NMR-based metabolomic approach here proposed, due to the short time of analyses and to lower costs compared to other analytical methods, was very useful for the study of seasonal variation of metabolites.

Indeed, although it is clear that secondary metabolites show peculiar trends, only fragmentary information is available, mainly because of the used approaches.

The most common and easiest procedure was to perform the phytochemical study on samples collected in a specific time of the year and then compare the other months (or seasons) by setting up a series of target analyses, often by HPLC [19, 20] or by less time and resource consuming approaches based on colorimetric assays [21].

The improvement of analytical methods, and especially the availability of a high-throughput approach like metabolomics [5], gives the chance to further explore the issue of seasonality [22] and to thoroughly study these changes.

Concerning the metabolites identified within the extracts, it is important to underline their biological activity.

Phenols are well known for their antioxidant activity [11]. As the studied plants are all very rich in phenolics, they have a great antioxidant potential. This could also explain the use of some of these herbs as natural food preservatives (Table 1).

Among the detected compounds, rosmarinic acid is the most widespread and the most abundant. A plethora of biological activities has been attributed to this compound, among them: adstringent, antioxidative, anti-inflammatory, antimutagen, antibacterial, and antiviral [23].

Many other phenylpropanoids and flavonoids were also detected. Several properties have been reported for these compounds, such as anti-inflammatory, antimicrobial, and antitumor activity [24].

The richness in these compounds of the studied plants supports their traditional uses.

However, the study evidenced that qualitative and quantitative variations of metabolites are observed along the year.

This is, to the best of our knowledge, the first report of metabolite content and seasonal qualitative and quantitative variation of this set of food spices. Furthermore, it is evidenced that a higher content of compounds known for their health promoting capacities can be found in spring and summer samples of all the analysed species, although season-specific compounds were also detected.

## 4. Conclusions

NMR-based metabolomics has been applied to the study of chemical composition of selected aromatic plants of Mediterranean vegetation.

The method allowed determining the chemical composition of plant extracts in terms of primary and secondary metabolites. The abundance of aromatic secondary metabolites suggested that the traditional uses of these plants might be supported by their chemical composition.

Furthermore, the seasonality of the accumulation of these metabolites was studied.

## Figures and Tables

**Table 1:** Studied plants.

Species and voucher specimen	Family	Uses
*Calamintha nepeta* L. CE236	Lamiaceae	Leaf used as food spice (usually added to meat, fish, and vegetable dishes; mint aroma) and for medicinal purposes (antiseptic, tonic, antispasmodic, diaphoretic, expectorant, etc.) [25, 26]

*Helichrysum italicum* G. Don CE233	Asteraceae	Leaf used as food spice (also known as “curry plant”) and for medicinal purposes (anti-inflammatory and anti-infective, antiallergic, etc.) essential oils used in cosmetics [16, 27]

*Foeniculum vulgare* Mill. CE237	Apiaceae	Leaf and fruits used to flavour several kinds of dishes. Also used in cosmetics and pharmaceutical products [28]

*Micromeria graeca* L. CE238	Lamiaceae	Leaf used as food spice (added to meat and vegetables)

*Origanum vulgare* L. CE239	Lamiaceae	Dried plant (epigeous part) used as food spice. The most common spice in Mediterranean cuisine. Used, since ancient times, for medicinal purposes (antioxidant digestive, expectorant, antiseptic, antispasmodic, etc.) [29]

*Satureja montana* L. CE234	Lamiaceae	Leaf used as food spice (usually added to meat, fish, and vegetable dishes). Natural food preservative. Savory honey is a very common ingredient in folk remedies. Used for medicinal purposes [30]

*Thymus longicaulis *C. Presl CE235	Lamiaceae	Leaf used as food spice (usually added to meat, fish, and vegetable dishes). Natural food preservative. Used also for medicinal purposes [31]

**Table 2:** Main metabolites detected in plant extracts. ^1^H-NMR data are measured in ppm and coupling constants (*J*) in Hertz. Relative amount is expressed as the mean value (*n* = 3) ± SD. For some metabolites, the quantitative analysis was not possible due to strongly overlapping signals; hence the presence is indicated by “X.”

Plant species	Metabolites	NMR	Wi	Sp	Su	Au
*Calamintha nepeta *	Alanine	1.48 (H3, d *J* = 7.2)	X	X	X	X
	Apigenin derivative 1^*^	6.51 (H6 d *J* = 2.1); 6.70 (H3 s); 6.78 (H8 d *J* = 2.1); 7.10 (H3′/H5′ d *J* = 8.7); 7.94 (H2′/H6′ d *J* = 8.7)		8.52 ± 1.08		5.25 ± 3.73
	Apigenin derivative 2^*^	6.54 (H6 d *J* = 2.1); 6.67 (H3 s); 6.73 (H8 d *J* = 2.1); 7.07 (H3′/H5′ d *J* = 8.7); 7.90 (H2′/H6′ d *J* = 8.7)				
	Apigenin derivative 3^*^	6.50 (H6 d *J* = 2.1); 6.66 (H3 s); 6.78 (H8 d *J* = 2.1); 7.02 (H3′/H5′ d *J* = 8.7); 7.88 (H2′/H6′ d *J* = 8.7)			7.81 ± 3.24	
	Apigenin derivative 4^*^	6.49 (H6 d *J* = 2.1); 6.61 (H3 s); 6.69 (H8 d *J* = 2.1); 7.06 (H3′/H5′ d *J* = 8.7); 7.84 (H2′/H6′ d *J* = 8.7)				
	Apigenin derivative 5^*^	6.55 (H6 d *J* = 2.1); 6.65 (H3 s); 6.69 (H8 d *J* = 2.1); 7.12 (H3′/H5′ d *J* = 8.7); 7.95 (H2′/H6′ d *J* = 8.7)	5.16 ± 0.59			
	Apigenin derivative 6^*^	6.53 (H6 d *J* = 2.1); 6.65 (H3 s); 6.66 (H8 d *J* = 2.1); 7.09 (H3′/H5′ d *J* = 8.7); 7.92 (H2′/H6′ d *J* = 8.7)				
	Citric acid	2.59 (H2a, d, *J* = 17.6); 2.72 (H2b, d, *J* = 17.6)	8.38 ± 3.46	13.66 ± 3.31	8.04 ± 0.17	13.44 ± 0.02
	Glucose	4.59 (H1 *β*, d, *J* = 7.8); 5.19 (H1 *α*, d, *J* = 3.8)	5.89 ± 2.33	6.87 ± 2.99	5.61 ± 0.28	5.64 ± 1.71
	Malic acid	2.39 (H3a, dd, *J* = 15.6, 9.3); 2.78 (H3a, dd, *J* = 15.6, 3.6); 4.31 (H2, dd, *J* = 9.3, 3.6)	26.72 ± 10.80		37.57 ± 2.72	31.45 ± 7.13
	Phenylpropanoid 1	5.97 (H8 d *J* = 15.9); 7.43 (H7 d *J* = 15.9)	3.04 ± 3.01	5.94 ± 0.45	3.05 ± 0.62	2.57 ± 0.67
	Phenylpropanoid 2	6.17 (H8 d *J* = 15.9); 7.30 (H7 d *J* = 15.9)	X			
	Phenylpropanoid 3	6.31 (H8 d *J* = 15.9); 7.52 (H7 d *J* = 15.9)	1.02 ± 0.99	4.62 ± 1.99	6.58 ± 1.51	
	Phenylpropanoid 4	6.45 (H8 d *J* = 15.9); 7.67 (H7 d *J* = 15.9)	X	X	X	X
	Phenylpropanoid 5	6.16 (H8 d *J* = 15.9); 7.25 (H7 d *J* = 15.9)		X		
	Phenylpropanoid 6	6.11 (H8 d *J* = 15.9); 7.37 (H7 d *J* = 15.9)			X	
	Phenylpropanoid 7	6.14 (H8 d *J* = 15.9); 7.39 (H7 d *J* = 15.9)				X
	Quinic acid	1.87 (H2a, m); 1.96 (H6a, m); 2.01 (H2b, m); 2.02 (H6b, m), 3.40 (H4, ov); 4.00 (H3, ov); 4.11 (H5, ov);	45.28 ± 6.65	55.09 ± 4.83	64.02 ± 4.16	71.61 ± 8.84
	Sucrose	4.15 (H3′, d, *J* = 8.4); 5.38 (H1, d, *J* = 3.6)	8.24 ± 3.63	7.09 ± 3.01	15.51 ± 2.84	10.37 ± 0.54

*Helichrysum Italicum *	Alanine	See *C. nepeta *	1.24 ± 0.19	0.87 ± 0.60	0.91 ± 0.15	1.51 ± 0.44
	Chlorogenic acid	1.84–2.20 (H2 and H6 quinic acid, m); 5.45 (H5, m); 6.37 (H8′, d, *J* = 15.9); 6.90 (H5′, d, *J* = 8.1); 7.07 (H6′, dd, *J* = 8.4, 2.1); 7.15 (H2′, d, *J* = 2.1); 7.62 (H7′, d, *J* = 15.9)	8.31 ± 1.55	9.03 ± 1.24	3.63 ± 2.59	3.26 ± 0.77
	Dicaffeoylquinic acid	6.30 (H8′, d, *J* = 16.2); 6.48 (H8′′, d, *J* = 15.6); 7.65 (H7′, d, *J* = 16.2); 7.66 (H7′′, d, *J* = 15.6)	10.86 ± 1.38	10.88 ± 5.16	11.53 ± 2.38	7.83 ± 2.07
	Glucose	See *C. nepeta *	3.77 ± 1.74	3.56 ± 0.04	2.73 ± 0.59	3.78 ± 2.01
	3-OH benzofuran	5.18 (H2 d *J* = 6.3); 5.22 (H3 d *J* = 6.3); 6.90 (H7 ov); 8.04 (H6 dd *J* = 8.4, 1.8)	4.06 ± 0.27	3.13 ± 1.39	5.37 ± 1.89	4.06 ± 1.47
	Isobenzofuranone	5.33 (H3 s); 6.73 (H4 d *J* = 1.8); 6.84 (H6 d *J* = 1.8)	6.03 ± 2.70			
	Malic acid	See *C. nepeta *	X	X	X	X
	Neochlorogenic acid	6.39 (H8′, d, *J* = 15.9); 7.52 (H7′, d, *J* = 15.9)	X	X	X	X
	Sucrose	See *C. nepeta *	16.93 ± 8.55	9.15 ± 4.57	10.24 ± 4.45	10.14 ± 3.75

*Foeniculum vulgare *	Alanine	See *C. nepeta *	1.17 ± 0.05	0.90 ± 0.49	1.24 ± 0.25	1.09 ± 0.09
	Caffeic acid	6.29 (H8′, d, *J* = 15.9); 6.88 (H5′, d, *J* = 8.1); 7.03 (H6′, dd, *J* = 8.4, 2.1); 7.12 (H2′, d, *J* = 2.1); 7.52 (H7′, d, *J* = 15.9)	X	X	X	X
	Chlorogenic acid	See *H. italicum *	X	X	X	X
	Dicaffeoylquinic acid	See *H. italicum *		X	X	X
	Glucose	See *C. nepeta *	1.17 ± 0.02	5.55 ± 2.89	5.77 ± 1.64	6.29 ± 1.38
	GABA (*γ*-aminobutyric acid)	1.92 (H3, m); 2.36 (H2, t, *J* = 7.5); 3.01 (H4, t, *J* = 7.5)	X	X		X
	Kaempferol	6.35 (H6, d, *J* = 2.1); 6.52 (H8, d, *J* = 2.1); 7.00 (H2′/H6′, d, *J* = 8.4); 8.09 (H3′/H5′, d, *J* = 8.4)	1.28 ± 0.17	1.78 ± 1.56	1.46 ± 0.44	1.18 ± 0.82
	Malic acid	See *C. nepeta *	44.68 ± 7.11	47.40 ± 10.23	168.52 ± 11.15	86.62 ± 3.49
	Quercetin	6.27 (H6, d, *J* = 2.1); 6.48 (H8, d, *J* = 2.1); 6.99 (H5′, d *J* = 8.5); 7.59 (H6′, d *J* = 8.5, 2.1); 7.75 (H2′, d *J* = 2.1)	1.93 ± 0.36	5.55 ± 3.61	6.23 ± 1.25	2.94 ± 2.09
	Quinic acid	See *C. nepeta *	25.09 ± 2.62	31.61 ± 17.48	33.49 ± 6.73	27.30 ± 2.25
	Sucrose	See *C. nepeta *	44.94 ± 2.28	36.80 ± 13.85	54.36 ± 11.86	30.62 ± 9.03
	Threonine	1.32 (H4, d, *J* = 6.6)	X	X	X	X

*Micromeria graeca *	Alanine	See *C. nepeta *	0.93 ± 0.55	1.40 ± 0.88	2.09 ± 0.89	5.75 ± 4.04
	Citric acid	See *C. nepeta *	19.89 ± 9.44	18.65 ± 1.56	17.13 ± 6.84	15.43 ± 5.10
	Glucose	See *C. nepeta *	4.81 ± 2.49	6.87 ± 2.99	5.61 ± 0.28	5.64 ± 1.71
	Malic acid	See *C. nepeta *	X	X	X	X
	Quinic acid	See *C. nepeta *	39.75 ± 10.68	55.09 ± 4.83	64.02 ± 4.16	57.41 ± 11.24
	Rosmarinic acid	3.00 (H7′a, dd, *J* = 14.1, 9.6); 3.15 (H7′b, dd, *J* = 14.1, 3.6); 5.02 (H8′, dd, *J* = 10.0, 3.3); 6.30 (H8, d, *J* =15.9); 6.71 (H6′, dd, *J* = 7.8, 2.1); 6.81 (H5′, d, *J* = 7.8); 6.82 (H5, d, *J* = 8.1); 6.89 (H2′, d, *J* = 2.1); 7.00 (H6, dd, *J* = 8.1, 1.8); 7.11 (H2, d, *J* = 1.8); 7.50 (H7, d, *J* = 15.9)	1.02 ± 0.99	4.62 ± 1.98	6.58 ± 1.51	1.54 ± 0.79
	Sucrose	See *C. nepeta *	7.99 ± 2.60	7.09 ± 3.02	15.51 ± 2.84	8.22 ± 3.73
	Threonine	See *F. vulgare *		X		

*Origanum vulgare *	Apigenin derivative 2	See *C. nepeta *	4.32 ± 0.24	2.95 ± 0.91		—
	Alanine	See *C. nepeta *	0.58 ± 0.12	0.90 ± 0.17	1.07 ± 0.14	—
	Choline	3.20 (s)	X	X		—
	Citric acid	See *C. nepeta *	14.74 ± 1.02	22.92 ± 4.13	15.54 ± 1.91	—
	Glucose	See *C. nepeta *	16.59 ± 2.64	3.62 ± 2.88	4.39 ± 3.82	—
	Lithospermic acid	3.00 (H7′a and b, ov) 6.30 (H8, d, *J* = 15.9); 7.82 (H7, d, *J* = 15.9)	X	X	X	—
	Malic acid	See *C. nepeta *	27.59 ± 1.69	32.40 ± 8.10	14.74 ± 8.51	—
	Quinic acid	See *C. nepeta *	30.94 ± 2.86	45.91 ± 1.83	43.12 ± 9.81	—
	Rosmarinic acid	See *M. graeca *	11.47 ± 6.11	15.73 ± 4.82	35.50 ± 5.91	—
	Sucrose	See *C. nepeta *	10.05 ± 1.58	4.98 ± 0.45	14.92 ± 4.36	—

*Satureja montana *	Apigenin derivative 5	See *C. nepeta *	X			
	Apigenin derivative 6	See *C. nepeta *	X			
	Alanine	See *C. nepeta *	X	X	X	X
	Choline	See *O. vulgare *	X	X		X
	Chlorogenic acid	See *H. italicum *	9.47 ± 1.20	15.01 ± 0.51	12.50 ± 5.54	3.66 ± 1.84
	Glucose	See *C. nepeta *	1.01 ± 0.67	3.96 ± 1.55	2.87 ± 1.47	4.27 ± 1.54
	Malic acid	See *C. nepeta *	39.32 ± 2.60	32.40 ± 0.99	8.55 ± 3.31	48.76 ± 6.78
	Rosmarinic acid	See *M. graeca *	7.15 ± 1.26	10.90 ± 1.64	8.84 ± 4.32	
	Sucrose	See *C. nepeta *	10.09 ± 0.93	8.56 ± 4.42	10.07 ± 1.25	9.37 ± 3.91

*Thymus longicaulis *	Alanine	See *C. nepeta *	0.34 ± 0.24	1.05 ± 0.11	1.08 ± 0.22	1.28 ± 0.07
	Citric acid	See *C. nepeta *	X	X	X	X
	Malic acid	See *C. nepeta *	26.92 ± 15.44	60.05 ± 3.28	35.56 ± 6.80	54.64 ± 14.72
	Quinic acid	See *C. nepeta *	19.85 ± 8.63	41.16 ± 7.50	40.71 ± 8.30	31.96 ± 3.17
	Glucose	See *C. nepeta *	3.06 ± 0.33	3.20 ± 0.83	2.65 ± 1.76	3.36 ± 1.41
	Phenylpropanoid 8	6.13 (H8 d *J* = 15.9); 7.46 (H7 d *J* = 15.9)	3.67 ± 1.13	5.65 ± 0.61	4.77 ± 1.24	1.90 ± 1.24
	Rosmarinic acid	See *M. graeca *	7.10 ± 3.70	7.78 ± 1.98	10.12 ± 1.76	4.87 ± 2.14
	Sucrose	See *C. nepeta *	7.10 ± 3.70	7.78 ± 1.98	10.12 ± 1.76	4.87 ± 2.14

Signal multiplicity indicated as follows: d = doublet, dd = doublet of doublets, m = multiplet, ov = overlapped, q = quartet, s = singlet, and t = triplet.

^*^Apigenin derivatives 1 and 2; 3 and 4; 5 and 6 were quantified together due to overlapping signals.
